# Impaired Metabolic Pathways Related to Colorectal Cancer Progression and Therapeutic Implications

**Published:** 2020-01

**Authors:** Chunyan QIU, Yue ZHANG, Longhua CHEN

**Affiliations:** 1 Department of Radiation Oncology, Nanfang Hospital, Southern Medical University, Guangzhou, China; 2 Department of Radiation Oncology, First Affiliated Hospital of Zhengzhou University, Zhengzhou, China

**Keywords:** Colorectal cancer, Metabolism, Short time series expression miner, Bioinformatics, Therapeutics

## Abstract

**Background:**

Risk of colorectal cancer (CRC) is defined by genetic predisposition and environmental factors that often co-occur and interact, resulting in diversiform biological reactions. The present study attempted to investigate transcriptome alteration and adaptation associated with CRC progression.

**Methods:**

The study consisted of patients who presented at Memorial Sloan-Kettering Cancer Center, Guangzhou, China with a colonic neoplasm in 1992–2004. Microarray GSE41258 of the study was acquired from Gene Expression Omnibus and 253 included microarrays were categorized by groups of normal colon, early primary tumor, lymph node metastases primary tumor, advanced primary tumor and distant metastases. Short Time-series Expression Miner (STEM) was applied to discover tumor grade-dependent gene expression patterns. Gene Ontology (GO) and Kyoto Encyclopedia of Genes and Genomes (KEGG) analyses were carried out to explore functional enrichment of differential expression genes (DEGs).

**Results:**

Overall, 2870 significant DEGs were screened out on all groups. Six significant grade-dependent gene expression patterns were statistically significant. DEGs in all significant patterns were mainly assembled in GO terms of metastases and deterioration of tumor, epithelial proteins and cytokines, and protein binding and bridging. DEGs in profile 0 down-regulated with higher tumor grade, prominently enriched in KEGG pathways of metabolism.

**Conclusion:**

Besides many well-known colorectal cancer-related pathways, DEGs of profiles especially those down-regulated with CRC progression, clustered in various metabolic pathways including starch and sucrose metabolism, fatty acid metabolism, nitrogen metabolism, as well as xenobiotics biotransformation that link to tumorigenesis, demonstrating the impairment of physiological metabolic pathways in the context of tumor progression. These results gave a high potential for therapeutic strategies.

## Introduction

Colorectal cancer (CRC) is the third most prevalent cancer of the globe and gives rise to the fourth-largest cancer-related death. Annually there are approximately 1.36 million raw cases and close to 70 million people died of this disease (1). Approximately 10% of CRC cases are hereditary, while up to 90% are sporadic (no family history or genetic predisposition) (2). Unlike other cancers, such as lung cancer, relative CRC risk is defined by genetic predisposition and environmental factors that often co-occur and interact: sociodemographic factors such as older age and male sex; medical factors such as family history, inflammatory bowel disease, diabetes; lifestyle factors such as smoking, obesity; diet factors such as high consumption of red and processed meat (3).

A classic colorectal cancer progression model was proposed considering the occurrence of tumor as “the outcome of the accumulation of obtained inherited and epigenetic changes that transform normal glandular epithelial cells into invasive adenocarcinomas”. Steps involve beginning the transformation from normal epithelium to benign neoplasia (adenoma), followed by invasive carcinoma, and eventually metastatic cancer (4). This series of events model, called “adenoma-carcinoma sequence”, often taking 10–15 years, is under numerous revisions with understanding of molecular pathogenesis improved. At present, at least four kinds of genomic or epigenetic instability mechanisms have been discovered in colorectal cancers: 1) chromosomal instability (CIN), 2) microsatellite instability (MSI), 3) CpG island methylator phenotype (CIMP), and 4) global DNA hypomethylation. To be specific, WNT signaling, TGF-β signaling, and epidermal growth factor receptor (EGFR) signaling are found to be the primary pathways that drive colorectal cancer (5). Our more comprehensive knowledge of molecular features on colorectal cancer has led to better diagnosis and treatment strategies, in particular, target therapies involving antibodies that target the VEGF and the EGFR(6).

In the procedure of colorectal cancer deterioration, tumor cells, in reality, are highly heterogenous and are continuously evolving. Therefore, we conducted the present study in an attempt to further delineate the transcriptome profile change and adjustment as a whole during the progression of colorectal cancer.

## Materials and Methods

### Microarray data acquisition and grouping.

Gene Expression Omnibus (GEO, http://www.ncbi.nlm.nih.gov/geo/) is a database repository of high throughput gene expression data and hybridization arrays, chips, microarrays supported by the National Center for Biotechnology Information (NCBI) at the National Library of Medicine (NLM). The Affymetrix microarray GSE41258 (7) taken in the platform of GPL96 (HG-U133A) Affymetrix Human Genome U133A Array was acquired from the GEO database. It was a biological specimens series consisted of primary colon adenocarcinomas, adenomas, metastasis and corresponding normal mucosae, which came from a study consist of patients presented at Memorial Sloan-Kettering Cancer Center with a colonic neoplasm from 1992 to 2004.

Altogether, 137 gene expression files were removed from the originally 390 Affymetrix genechips, including17 repeats, 2 outliers with high rates of absent values, 9 normal colon samples whose expression profiles were markedly different from the other normals, 2 mislabeled samples, 1 microadenoma, 2 high-grade adenomas, 30 noncolon normal samples and cell lines, and 28 metastasis samples that exhibited high levels of stromal contamination, and 46 polyps (7). After filtering, 253 samples were included: 43 normal colon epithelia, 180 primary carcinomas (28 clinical stage I, 47 clinical-stage II, 49 clinical-stage III, 56 clinical-stage IV), 21 liver metastases, and 9 lung metastases. Regarding to different biological feature of various tumor stages, we categorized them into five prominent groups: normal colon (43 microarrays), early primary tumor (28 stage I and 47 stage II), lymph node metastases primary tumor (49 stage III, “LNM” for short), advanced primary tumor (56 stage IV) and distant metastases (21 liver metastases and 9 lung metastases).

### Data pre-processing and DEGs screening.

Raw data of .cel file was extracted and then subjected to Affymetrix MAS5 algorithm to get background adjustment, normalization and summarization through R affy package (http://www.bioconductor.org/packages/release/bioc/html/affy.html). Differential expression genes (DEGs) were defined as genes with |log_2_ fold change (FC)|≥1 and a false detection rate (FDR) <0.05 between normal colon and early, LNM, advanced and metastases tumor samples, respectively.

### Bioinformatics analysis

**[1] Grade-dependent patterns clustering.** First, we combined all the DEGs in four compared groups. Gene expression pattern analysis was performed on all DEGs by Short Time-series Expression Miner software(STEM) (8) on the OmicShare tools, a free online platform for data analysis and plotting (www.omicshare.com/tools). STEM is the first software application designed for the gene expression trending analysis of short time-series datasets (3–8 time points).

Parameters were set as follows: 1) Maximum Unit Change in model profiles between time points is 1; 2) Maximum output profiles number is 20 (similar profiles will be merged); 3) Minimum ratio of fold change of DEGs is no less than 2; 4) Significant level was set to be ≤0.05.

**[2] Functional clustering.** The Gene Ontology (GO) project was to provide controlled vocabularies for the description of the biological process, molecular function, and cellular component of gene products (9). Kyoto Encyclopedia of Genes and Genomes (KEGG) is a collection of manually drawn KEGG pathway maps representing experimental knowledge on metabolism and other functions of the cell and the organism, designed to link genes in the genome to gene products (i.e. proteins) in the pathway (10). GO and KEGG pathway enrichment analyses were performed using the OmicShare tools as following steps: DEGs were mapped to GO terms in the Gene Ontology database (http://www.geneontology.org/)(11) or pathways in KEGG database (http://www.genome.jp/kegg/) (12), significantly enriched GO terms or pathways in DEGs comparing to the genome background were deined by hypergeometric test. The calculated *P-*value was gone through FDR correction, taking FDR ≤ 0.05 as a threshold.

## Results

### DEGs screening.

**Fig. 1:**
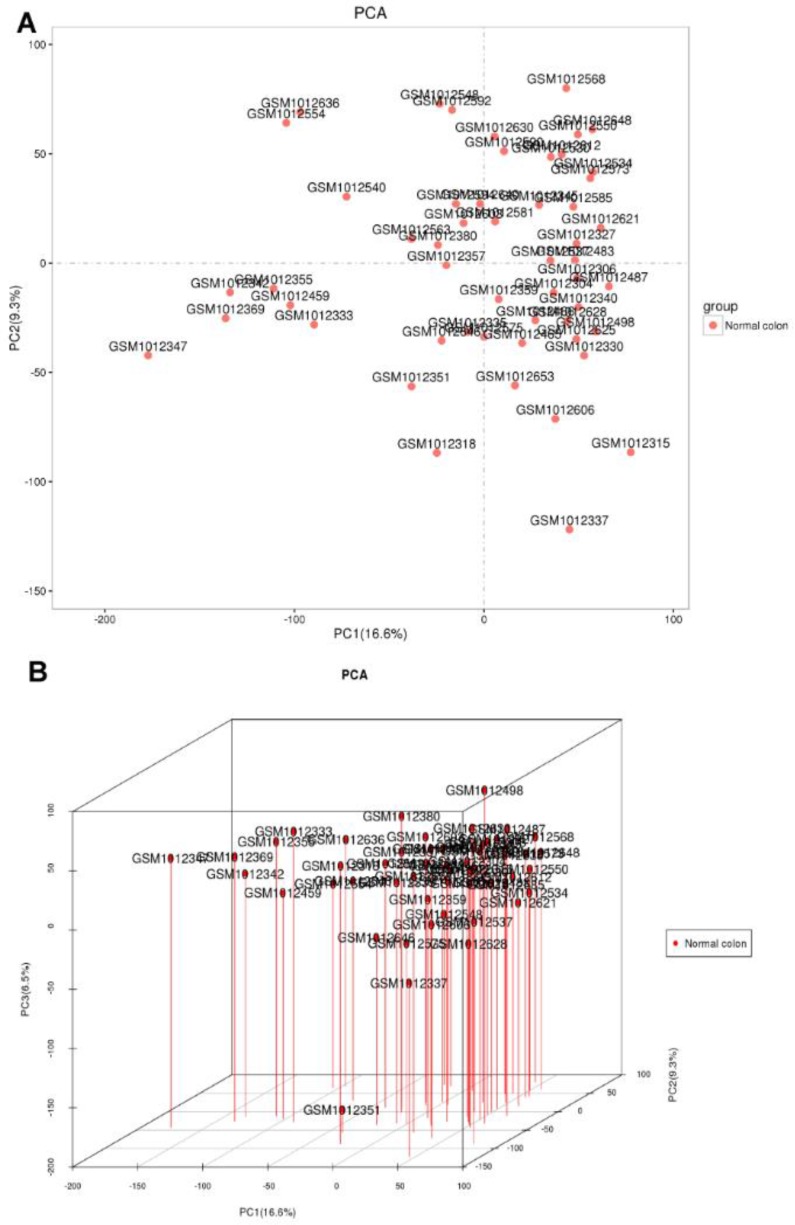
Filtering of samples. (A). 2D-PCA for samples. (B). 3D-PCA for samples. Excluded samples: GSM1012347, GSM1012342, GSM1012369, GSM1012355, GSM1012459, GSM1012333, GSM1012554, GSM1012636, GSM1012540, GSM1012351

In total, 2870 significant DEGs were screened out, among which 1668 in comparison between early-stage and normal colon, 1618 between lymph node metastasis and normal colon, 1624 between advanced stage and normal colon, 2308 between distant metastases and normal colon. Detailed overlaps between each comparison pair were illustrated in Fig. 1.

### Grade-dependent patterns clustering.

**Fig. 2:**
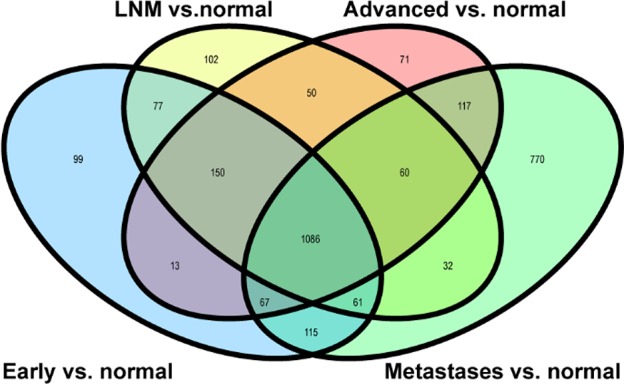
Differential expression genes overlap. It shows a venn diagram of DEGs in four compared groups: early stage vs. normal; lymph node metastasis stage vs. normal; advanced stage vs. normal; metastases vs. normal

In order to discover tumor grade-dependent gene expression pattern, we applied the Short Time-series Expression Miner (STEM). While not all 2870 DEGs were detectable in every group, the OmicShare tools platform modified STEM software by automatically recoding those assigned expression value 0 to 0.001, avoiding these genes being filtered. Consequently, 20 expression pattern profiles were generated, among which profile 0, 19, 18, 2, 5, 3 were statistically significant with an order of increasing *P*-value. The above significant profiles were comprised of 737, 552, 469, 259, 87 and 63 DEGs, respectively (Fig. 2A). As shown in Fig. 2B, genes in profile 0 showed a decrease expression trend when tumor grade getting higher, whereas genes in profile 19 presented a reverse trend. A gene expression process of elevation, stableness and then decline was observed in profile 18. Gene expression patterns seemed much similar in profile 2, 5 and 3, where levels of expression went through former down-regulation and latter up-regulation.

### GO terms enrichment.

As Table 1 demonstrated, taking FDR≤0.05 as significant level, genes in all significant profiles were enriched in GO terms of biological process as follows: extracellular matrix organization, extracellular structure organization, movement of cell or subcellular component, cell motility, localization of cell, localization, cell proliferation and locomotion. Regarding GO terms of cellular component, genes were clustered in extracellular region part, extracellular space, extracellular region, which were mostly epithelial genes or cytokine genes including CD40LG, SPON1, SERPINB5, TPSAB1, CCL3L1, CCL3L3, CXCL11, COL1A1, COL11A1, MATN3, CCL23, CCL5, APOC1, F13A1, MMP28, INHBA, CCL18, BMP8A, PYY, etc. As for the aspect of GO terms of molecular function, genes were clustered in protein binding and bridging, binding and bridging.

**Table 1:** GO terms enrichment for DEGs of the 6 significant profiles

***GO ID***	***Description***	***Target Gene No.(71)***	***Ref Gene No.(19693)***	***FDR***
Biological Process				
GO:0030198	extracellular matrix organization	10	406	0.000231
GO:0043062	extracellular structure organization	10	406	0.000231
GO:0006928	movement of cell or subcellular component	15	1334	0.003785
GO:0048870	cell motility	15	1334	0.003785
GO:0051674	localization of cell	15	1334	0.003785
GO:0051179	localization	35	5570	0.010621
GO:0008283	cell proliferation	18	2037	0.010621
GO:0040011	locomotion	17	1910	0.012753
Cellular Component				
GO:0044421	extracellular region part	19	1658	0.000244
GO:0005615	extracellular space	16	1468	0.001535
GO:0005576	extracellular region	32	4844	0.002534
Molecular Function				
GO:0030674	protein binding, bridging	5	162	0.008804
GO:0060090	binding, bridging	5	162	0.008804

### KEGG pathway enrichment.

**Fig. 3:**
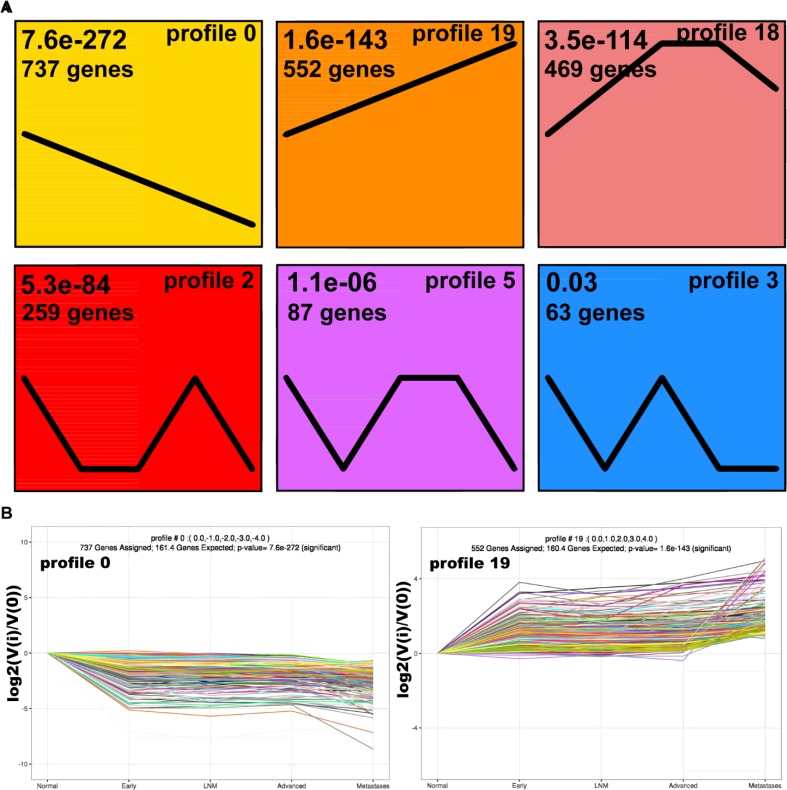
Grade-dependent pattern clustering by short time series expression miner analysis. (A). Statistically significant profiles of 20 output profiles. Each box corresponds to a signifcant expression pattern. The number at the top represents DEGs number clustered in corresponding pattern. The number in the upper left represents the changing P-value. (B). Gene expression changes in the two most signifcant profiles at different tumor stages. Each line in the fgure represents an expression value of the corresponding gene. The abscissa represents the grades of tumor, and the ordinate represents the log2 value of the fold change. Negative values indicate downregulated expression, while positive values indicate upregulated expression

**Fig. 4:**
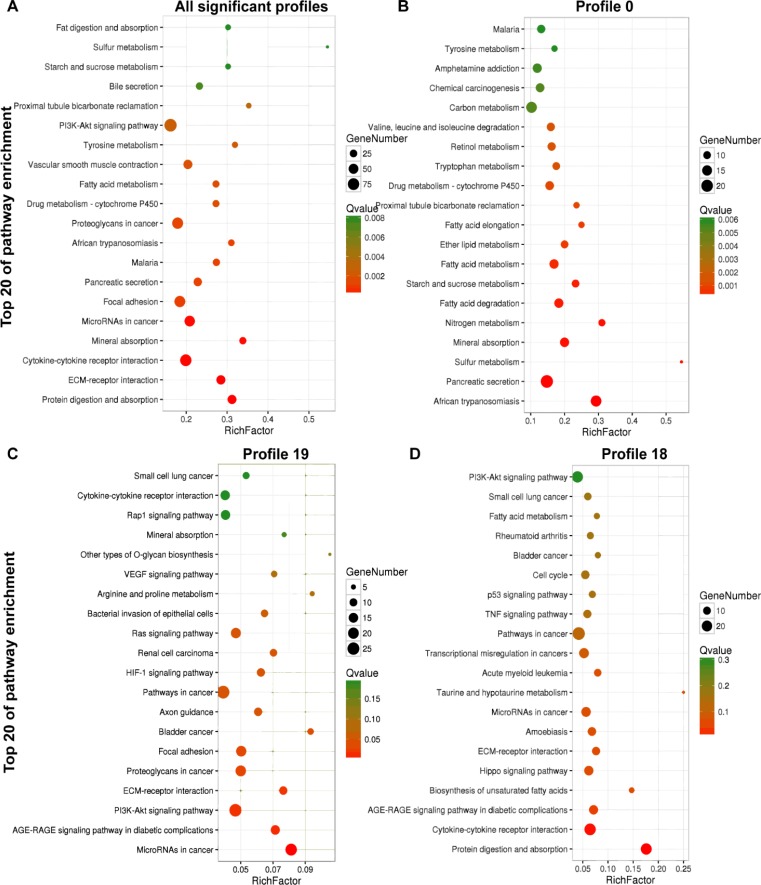
KEGG pathway enrichment. (A) Bubble chart of KEGG pathway enrichment for DEGs in the 6 significant profiles. (B) Bubble chart of KEGG pathway enrichment for DEGs in profile 0. (C) Bubble chart of KEGG pathway enrichment for DEGs in profile 19. (D) Bubble chart of KEGG pathway enrichment for DEGs in profile 18. The chart shows a grid where Y-axis represents an enriched KEGG pathway of the top 20(significant levels become lower from the bottom to the top) and X-axis the rich factor which means target genes number divided by reference genes that enriched in each pathway. While, each cell reports a bubble whose size indicates the number of genes enriched and color indicates the Q value

For DEGs in all significant profiles, the top 20 enrichment KEGG pathways were composed of the following classes: digestive system, signaling molecules and interaction, cancers, infectious diseases, metabolism, circulatory system as well as excretory system (Fig. 3A). While for DEGs in profile 0 whose expression level turned down in higher tumor grade, the top 20 enrichment KEGG pathways were mainly metabolism pathways, take it for example, nitrogen metabolism, sulfur metabolism, starch and sucrose metabolism, fatty acid metabolism, ether lipid metabolism, etc. (Fig. 3B). For DEGs in profile 19 whose expression level increased with higher tumor grade, the top 20 enrichment KEGG path- ways mostly aggregated in well-known carcinogenesis pathways including microRNAs in cancer (13), PI3K-Akt signaling pathway (14), ECM-receptor interaction (15), proteoglycans in cancer (16), focal adhesion (17), HIF-1 signaling pathway (18), VEGF signaling pathway (19), cytokine-cytokine receptor interaction (20) etc. (Fig. 4A).

KEGG pathway enrichment for DEGs in profile 18 seemed to distribute in many different aspects (Fig. 4B). Since DEGs in profile 2, 3, and 5 shared a similar expression pattern, we integrated these DEGs to perform KEGG pathway enrichment analysis but not any result was generated.

## Discussion

In this gene transcriptome study, we made comprehensive analyses on 253 microarrays grouped by normal colon, early-stage, lymph node metastasis, advanced primary colorectal cancer, and metastases samples. By implementing the Short Time-series Expression Miner (STEM), we discovered six significant grade-dependent gene expression patterns. The above mentioned GO terms for DEGs in all significant profiles were highly associated with metastases and aggressiveness of carcinoma of numerous various types (21–23). Some of the enriched epithelial genes or cytokine genes have been reported to get involved in cancer development and progression, for example, SERPINB5 in breast cancer (24), CCL3L1 in glioblastoma (25), CXC group in lung cancer (26), MMP28 in gastric cancer (27) and so on. On the other hand, DEGs of all significant grade-dependent profiles were clustered in diverse KEGG pathways. Interestingly, we found that for DEGs in profile 0 down-regulated expressed with higher tumor grade, the top 20 KEGG pathways concentrated in metabolism pathways. Such results highlight the disrupted physiological metabolic pathways in the context of tumor progression.

As early as in 1920s, German biochemist Otto Warburg recognized an unusual metabolic phenomenon of cancer cells, called “Warburg effect”, also known as aerobic glycolysis rather than oxidative phosphorylation in normal cells, is defined as a high rate of glucose utilization and lactate production despite the presence of sufficient oxygen to oxidize glucose carbon in the mitochondria (28). Another metabolic feature of tumor cells is the increased fatty acid synthesis, and the occurrence and progression of tumors are related to the de novo synthesis of lipopolysaccharides in their individual cells. Normally, cells mainly utilize fatty acids from the extracellular intake to converse into triglycerides, in order to store energy in the body. However, in spite of abundant supply of exogenous fatty acids, tumor cells rarely use them but rely on glycolysis products pyruvate to synthesise endogenous fatty acid (29). Although the detailed mechanism remained unclear, as is supported by our study results, down-regulated genes in starch and sucrose metabolism pathway or carbon metabolism pathway such as UGT1A and UGT2B (30), EHHADH (31), SHMT1 (32), ACAT1 (33), ACADS (31), ACADM (34) was previously reported to link with CRC. In addition, more implication was observed in fatty acid metabolism, fatty acid degradation, and fatty acid elongation pathway as many of their clustered DEGs were found to be potentially related to CRC oncogenesis or patient progression, such as EHHADH (31), ACAA2 (34), CPT2 (34), ACAT1 (33), ACADM (34), ELOVL4 (35), which reflected the damage of normal fatty acid intake and transformation and utilization pathway.

**Fig. 5:**
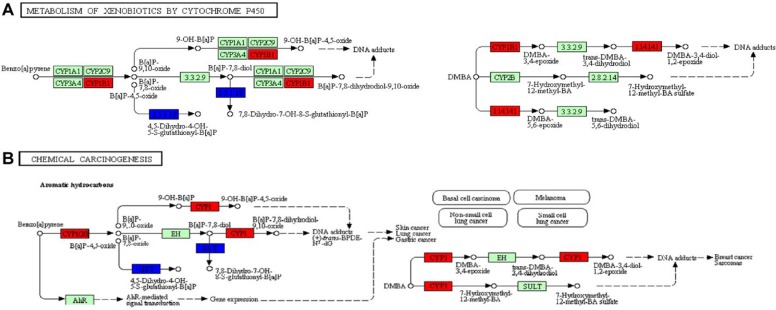
Biotransformation and carcinogenesis. (A). Part of hsa00980: metabolism of xenobiotics by cytochrome P450. (B). Part of hsa05204: chemical carcinogenesis. Red color indicates DEGs in profile 19 and blue color indicates DEGs in profile 0

Our results also showed alteration of biotransformation pathways. Biotransformation defined as processes of all modifications that alter the structure of chemicals is critical to chemical carcinogenesis of CRC. Biotransformation reactions consist of two distinct stages. Phase I reactions involve the introduction of polar functional groups into the molecule or modification by oxidation, reduction or hydrolysis. Phase II reactions comprise chemical conjugations to water-soluble molecules. Generally, phase II reactions reduce the toxicity, while phase I biotransformation usually activates chemical compounds (36). Cytochrome P450 (CYP) superfamily are the most important phase I metabolic enzymes, while Glutathione-S-Transferases (GST), N-acetyltransferases (NAT), Sulfotransferase (SULT) and UDP-glucuronosyltransferase (UGT) are crucial components in the phase II metabolism. Our study revealed that during the process of xenobiotics metabolism and chemical carcinogenesis, exemplarily, for cancerogenic benzopyrene and DMBA, cytochrome P450 tend to be up-regulated while phase II metabolic enzymes such as GST, NAT and UGT are usually down-regulated (Fig. 5). With deterioration of CRC, the capacity of cells to metabolize xenobiotics especially carcinogenic compounds is hampered to a great extent. These supported the previous findings that gene polymorphism of CYP1A1 (37), CYP1A2 (38), and CYP2E1 (39) were highly associated with CRC. Besides, the high activity of phase II metabolic enzymes including GST superfamily (GSTM1 and GSTT1 (40)), SULT superfamily (SULT1A1 (41)) and UGT superfamily (UGT1 and UGT2 (42)) was reported to more or less protect against dietary and/or environmental chemicals involved in the pathogenesis of colorectal cancer.

A number of researches began to focus on the metabolic pathway as potential targets for treating CRC. A mechanism was established by which the miR-1/Smad3/HIF-1α axis facilitates the Warburg effect to promote CRC progression in vitro and in vivo, showing the potential role of miR-1 in molecular therapy of patients with advanced colorectal cancer (43). As for lipid metabolism pathways, targeted inhibition of lipogenic enzymes abolished expression of CD44, a transmembrane protein associated with metastases in several cancers including CRC, as well as attenuated the activation of MET, Akt, FAK, and paxillin, known to regulate adhesion, migration, and invasion. Such consequences demonstrated that novel lipogenesis may be a promising therapy clue for advanced CRC (44). While the presence of IL6 linked to neoplastic tissue can modify metabolic competency of colon epithelial cells (45). This is achieved through the modulation of CYP1B1 expression and CYP2E1 in epigenetic and transcriptional scale, resulting in more activation of dietary carcinogens and DNA damage, which promotes colorectal carcinogenesis ultimately. Likewise, the siRNA-mediated inhibition of β-catenin signaling, aberrantly activated in a majority of colorectal cancers, modulated genotoxicity of dietary carcinogen BaP in colon cell model in vitro, via a mechanism involving up-regulation of CYP1 expression and activity (46). Although many such studies are currently in an early stage far from clinical application, with some elucidating the detailed mechanisms of altered metabolic pathways functioning in exacerbation of CRC, and some trying to inhibit these pro-tumor pathways in vitro and in vivo with agents, targeting carcinogenic metabolic pathways have brought promising therapeutic strategies. On the other hand, with more understanding achieved on the immense effect of gut microbiome on the modulation of host metabolism to environmental exposure in recent years, more advanced researches combining transcriptomics, proteomics as well as metabolomics is necessary to acquire further understanding of metabolic alteration and adaptation during tumor exacerbation (47).

By providing insight into integrated biological metabolic alteration and adaptation during progression of colorectal cancer, we are to facilitate a better understanding of tumor progression and discovery of novel therapeutic strategy with accounting pathways concerned. Certainly, further studies are needed to pave in this way.

## Conclusion

DEGs in all significant patterns were mainly assembled in GO terms of metastases and deterioration of tumor, epithelial proteins and cytokines, and protein binding and bridging. DEGs down-regulated with higher tumor grade were found to be closely linked to KEGG pathways of metabolism. Further, metabolic pathways related to colorectal cancer progression such as aerobic glycolysis, increased fatty acid synthesis, aberrant bio-transformation presented high potential for therapeutic strategies.

## Ethical considerations

Ethical issues (Including plagiarism, informed consent, misconduct, data fabrication and/or falsification, double publication and/or submission, redundancy, etc.) have been completely observed by the authors.
